# Evaluating the Strength and Impact of Raw Ingredients of Cement Mortar Incorporating Waste Glass Powder Using Machine Learning and SHapley Additive ExPlanations (SHAP) Methods

**DOI:** 10.3390/ma15207344

**Published:** 2022-10-20

**Authors:** Hassan Ali Alkadhim, Muhammad Nasir Amin, Waqas Ahmad, Kaffayatullah Khan, Sohaib Nazar, Muhammad Iftikhar Faraz, Muhammad Imran

**Affiliations:** 1 Department of Civil and Environmental Engineering, College of Engineering, King Faisal University, Al-Ahsa 31982, Saudi Arabia; 2 Department of Civil Engineering, COMSATS University Islamabad, Abbottabad 22060, Pakistan; 3 Department of Mechanical Engineering, College of Engineering, King Faisal University, Al-Ahsa 31982, Saudi Arabia; 4 School of Civil and Environmental Engineering (SCEE), National University of Sciences & Technology (NUST), Islamabad 44000, Pakistan

**Keywords:** cement mortar, waste glass powder, building material, compressive strength, flexural strength

## Abstract

This research employed machine learning (ML) and SHapley Additive ExPlanations (SHAP) methods to assess the strength and impact of raw ingredients of cement mortar (CM) incorporated with waste glass powder (WGP). The data required for this study were generated using an experimental approach. Two ML methods were employed, i.e., gradient boosting and random forest, for compressive strength (CS) and flexural strength (FS) estimation. The performance of ML approaches was evaluated by comparing the coefficient of determination (R^2^), statistical checks, k-fold assessment, and analyzing the variation between experimental and estimated strength. The results of the ML-based modeling approaches revealed that the gradient boosting model had a good degree of precision, but the random forest model predicted the strength of the WGP-based CM with a greater degree of precision for CS and FS prediction. The SHAP analysis revealed that fine aggregate was a critical raw material, with a stronger negative link to the strength of the material, whereas WGP and cement had a greater positive effect on the strength of CM. Utilizing such approaches will benefit the building sector by supporting the progress of rapid and inexpensive approaches for identifying material attributes and the impact of raw ingredients.

## 1. Introduction

Several practices, including manufacturing, mining, electricity generation, steel and iron metallurgy, production of electronic devices, etc., result in large volumes of solid waste [1]. Numerous harmful wastes are combustible, caustic, ignitable, virulent, and chemically reactive, and their dumping in landfills has caused substantial economic losses [2,3]. Therefore, it is desirable to recycle or reuse the solid waste in building materials [4,5,6]. In the building sector, cement mortar (CM) is commonly used [7,8,9]. Various strategies have been adopted by researchers to enhance the performance of CM [10,11]. For example, to improve the performance of CM, waste materials may be utilized as an alternative to aggregate [12,13], reinforcing fibers [14,15], and cement substitutes [16,17]. Due to the partial replacement of aggregates and cement, natural resources may be conserved, and CO_2_ emissions might decrease [18,19,20]. It has also been noticed that using some waste materials enhances the performance of CM [21,22]. Globally, a substantial amount of glass waste (GW) is generated, with a substantial volume of GW discarded in landfills [23]. Although a growing number of towns are producing more municipal solid waste, landfill space is becoming gradually insufficient, especially in large cities [24]. Compared to other wastes (plastic and wood), GW is more chemically stable. Long-term burial of GW does not render it biodegradable [25]. Moreover, certain types of glass, such as cathode ray tubes (CRT), contain toxic elements, such as lead, cadmium, mercury, and beryllium, polluting underground soil and water [26]. China generates over 43 million tons of CRT glass annually [27], posing major environmental risks and endangering human health. GW may be recycled, crushed and utilized to partly substitute aggregates and cement in CM [28,29,30]. Prior research has demonstrated that GW recycling in CM is the most effective method [28,29]. Thus, the use of GW as a substitute for aggregate in construction materials will preserve natural resources and facilitate waste management. In the same way, incorporating GW as a cement substitute will help decrease cement requirements and, consequently, CO_2_ emissions [31,32].

Numerous experiments have been conducted to assess the characteristics of CM and compressive strength (CS) is interpreted as significant [18]. The CS of the CM provides crucial information on its various properties. The CS of concrete is connected, either directly or indirectly, to a number of mechanical and durability properties [33]. Similarly, flexural strength (FS) is an essential component that must be considered in the design of CM structures, as it affects deflection characteristics, flexural cracking, brittleness ratio, and shear strength [34]. Currently, analytical models for the strength of materials are being developed in an effort to eliminate tests and resources that are unneeded. Various traditional models, including best-fit curves, are used to simulate the material characteristics (developed using regression analysis). Due to the nonlinear nature of CM [35,36], conventional regression approaches may not effectively represent the material’s inherent behavior. In addition, regression methods may exaggerate the significance of some variables [37]. Methods based on artificial intelligence, such as supervised machine learning (ML), are amongst the most highly advanced modeling approaches employed in the current topic area [38,39,40,41,42]. These approaches simulate reactions based on input characteristics, and testing validates the yield models. The use of ML approaches to predict the properties of CM and bituminous blends is gaining popularity [43,44,45]. The bulk of prior ML-based investigations focused on forecasting the strength of conventional CM [46,47,48], whereas just a handful focused on predicting the properties of CM that integrated GW. Therefore, further ML-based studies are required to examine the validity of ML techniques on CM that contains WG. Moreover, it is challenging to investigate the impact of several raw ingredients on the material strength in a single study using experimental methods. Thus, it is required to apply advanced methods to examine the impact of various raw ingredients on the material strength in a fast and efficient manner.

This work utilized experimental data to assess the CS and FS of CM with waste glass powder (WGP), used to partially replace cement and fine aggregate, by employing ML methods. Samples of CM were cast using varied quantities of WGP as cement and fine aggregate replacements from 0 to 15%, with an increment of 2.5%. The CS and FS of the samples that contained WGP were recorded at 28 days of age. Two ML methods were employed, i.e., random forest regressor (RFR) and gradient boosting regressor (GBR), to achieve the study’s aims. Both GBR and RFR are ensemble ML methods [49]. Because it is obvious from the literature that ensemble ML approaches outperform individual ML methods [45,50], only ensemble ML methods are selected in this research to determine the best predictor. The performance evaluation of each model was carried out and compared by the coefficient of determination (R^2^), statistical checks, k-fold method, and variation in the predicted results (errors). Experimental-based studies entail extensive efforts, expenditures, and time for material collection, sample casting, curing, and performing tests. By decreasing these efforts using advanced techniques such as ML, the construction sector will benefit as a result. Due to the fact that several raw ingredients, such as cement, fine aggregate, water, silica fume, superplasticizer, and WGP, influence the strength of CM, it is difficult to measure their combined influence using experimental methods. In this instance, a SHapley Additive ExPlanations (SHAP) analysis was performed to explore the interaction and impact of raw ingredients on the CS and FS of CM. A data sample is needed for ML techniques, which can be generated from the experimental approach. The data generated may consequently be employed to train ML techniques and approximate material properties. The present study used 6 input parameters to foretell the CS and FS of WGP-based CM and assess the efficacy of each ML approach and the impact of raw ingredients on its strength.

## 2. Research Methods

### 2.1. Dataset Used for Modeling

**Figure 1:**
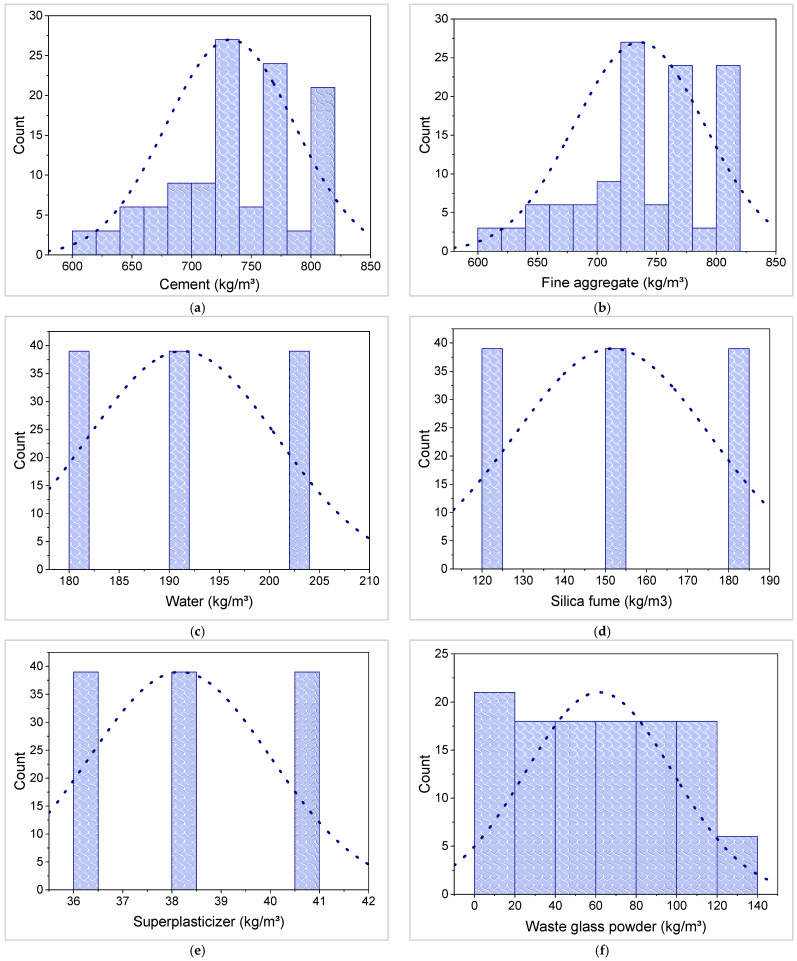

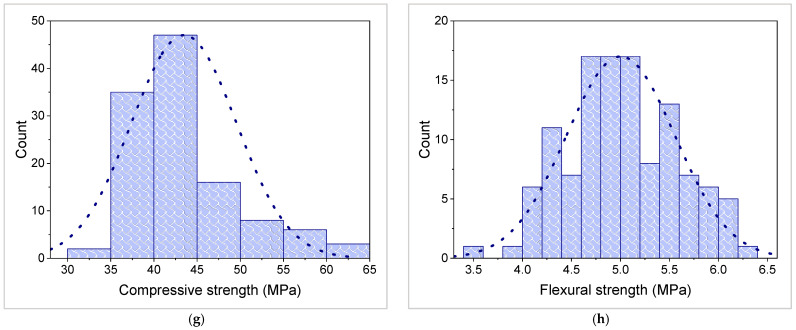
Relative frequency dispersal of input and output parameters: (**a**) Cement; (**b**) Fine aggregate; (**c**) Water; (**d**) Silica fume; (**e**) Superplasticizer; (**f**) Waste glass powder; (**g**) Compressive strength; (**h**) Flexural strength.

In order to obtain the desired results, ML approaches require a broad collection of input parameters [51]. In this regard, an experimental study was carried out using six raw ingredients, including cement, fine aggregate (FA), water, silica fume (SF), superplasticizer (SP), and WGP. Samples of CM were cast with varying amounts of WGP as a cement and fine aggregate replacement, ranging from 0% to 15% with a 2.5% increment. Compressive and flexural strength tests were performed on the samples of 50 mm cubes and 40 mm × 40 mm × 160 mm prisms, respectively, after 28 days of water curing to assess the CS and FS. In this study, data sample was generated from the experimental work and used to train ML models. All six raw ingredients were taken as inputs, and CS and FS as the outputs for ML-based modeling. Table 1 provides the descriptive statistics for all inputs and outputs utilized for modeling. Mode, median, and mean illustrate the fundamental tendencies, whereas standard deviation, minimum, and maximum highlight variation. The frequency dispersion of each input and output component is provided in Figure 1.

### 2.2. Machine Learning-Based Modeling

**Figure 2:**
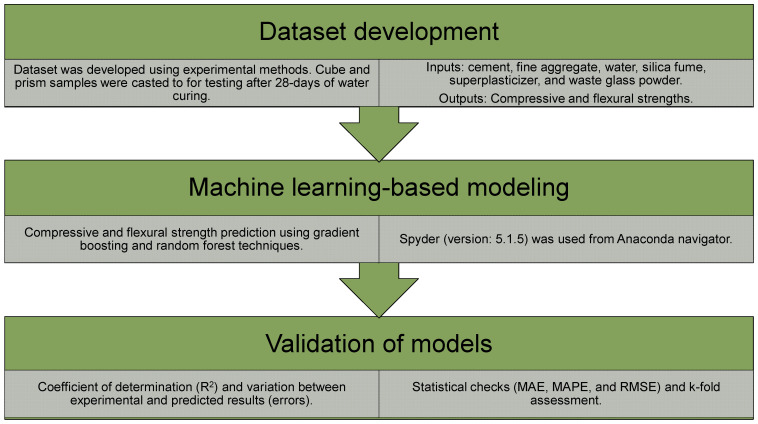
Sequence of dataset development, modeling, and validation methods.

Using the experimental data, the CS of CM that contained WGP was evaluated. The procedures utilized cement, fine aggregate, water, silica fume, superplasticizer, and WGP as inputs, with CS and FS serving as the outputs. Ensemble ML approaches with Python code, and Anaconda Navigator software were utilized to achieve the aims of the research. The ML models were operated using Spyder (version 5.1.5). GBR and RFR ML methods were used to assess the CS and FS of WGP-based CM. These ML algorithms are generally used to estimate required results using input variables. These approaches may be used to forecast a material’s strength, temperature resistance, and durability [52,53]. During the modeling phase, six input characteristics and two outputs (CS and FS) were used. The proportion of experimental data used for modeling was 30% for testing and 70% for validation. The R^2^ value of the expected outcome indicates the exactness of a model. The R^2^ value reveals the amount of deviation; a number close to zero indicates greater variation, whereas a number close to one suggests that the prediction model and experimental results are almost entirely matched [54]. On both models, k-fold, statistical, and error assessments, including mean absolute error (MAE), mean absolute percentage error (MAPE), and root mean square error (RMSE), were performed. Figure 2 demonstrates the sequence of the modeling techniques. The succeeding subsections describe the ML methods and validation approaches utilized in this work.

#### 2.2.1. Gradient Boosting Regressor

**Figure 3:**
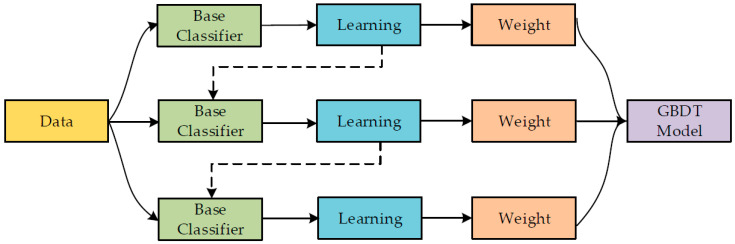
Sequence of gradient boosting regressor training procedure [56].

Friedman [55] suggested this ensemble approach for classification and regression. GBR is similar to other boosting approaches, but is restricted to regression alone. As observed in Figure 3, each training set repetition is picked at random and verified by the base model in this approach. GBR’s accuracy and speed may be improved by randomly subsampling the training data, which eventually prevents overfitting. The lower the amount of training data samples, the greater the rate of regression to fit. GBR requires the shrinkage rate and n-trees tuning variables, where n-trees represents the figure of trees generated. Here, the number of n trees should not be too low, and the shrinkage factor, also known as the learning rate, applies to each expansion tree.

#### 2.2.2. Random Forest Regressor

**Figure 4:**
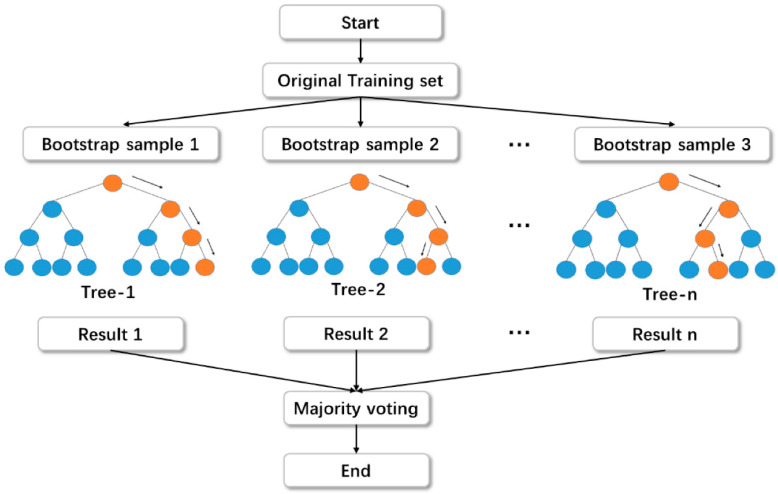
Structure of random forest-based modeling [59].

RFR is accomplished by means of random split selection on bagged decision trees [57]. The construction and procedure of the RFR model are depicted schematically in Figure 4. Each tree in the forest is generated by utilizing an arbitrarily selected training set, and each split within a tree is constructed by utilizing an arbitrarily chosen subgroup of input parameters, developing a forest of trees [58]. This element of uncertainty increases the tree’s variety. The forest is comprised solely of mature binary trees. The RFR approach has shown to be a highly successful regression tool for general applications. The approach, which accumulates the calculations of many randomized decision trees, has demonstrated greater precision when the number of variables surpasses the number of interpretations. In addition, it is adaptable to both large-scale and ad hoc learning tasks, delivering metrics of varying significance [57].

#### 2.2.3. Validation of Machine Learning-Based Models

**Figure 5:**
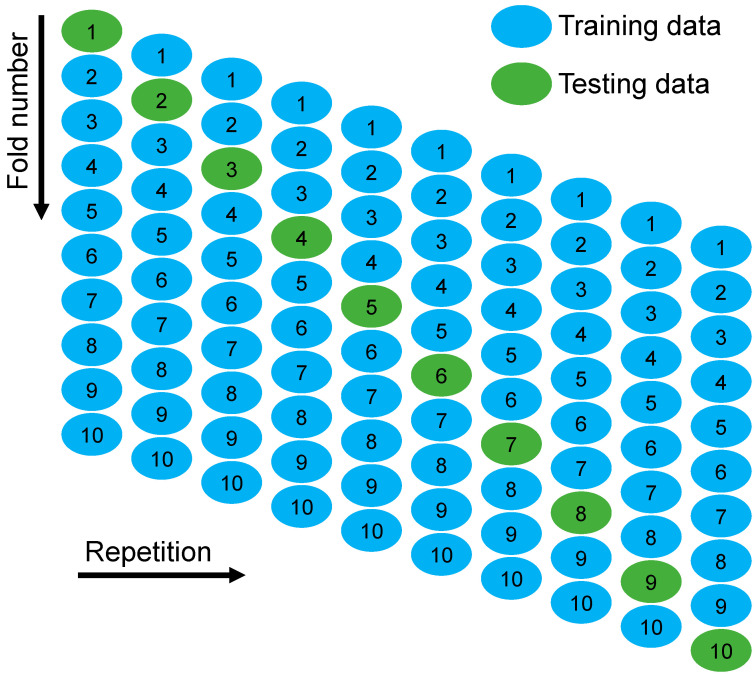
Schematic image of the k-fold approach [63].

Statistical checks and k-fold techniques were adopted to confirm the deployed ML algorithms. Typically, the k-fold method is employed to evaluate the effectiveness of a model by randomly splitting relevant data samples into 10 classes [60]. As shown in Figure 5, nine classes are used to train ML models, whereas only one is used for validation. The ML approach is more accurate when errors are smaller and R^2^ is larger. In addition, the desired result requires 10 repeats of the technique. This effort greatly adds to the model’s outstanding accuracy. In addition, each ML technique’s precision was statistically evaluated using error evaluation (MAE, MEPE, and RMSE). The accuracy of the ML methods’ projections was statistically evaluated using Equations (1)–(3) derived from past research [61,62].

(1) $$\mathrm{MAE}=\frac{1}{n}\sum_{i=1}^{n}\left|P_{i}-T_{i}\right|,$$

(2) $$\mathrm{RMSE}=\sqrt{\sum \frac{{\left(P_{i}-T_{i}\right)}^{2}}{n}},$$

(3) $$\mathrm{MAPE}=\frac{100\%}{n}\sum_{i=1}^{n}\frac{\left|P_{i}-T_{i}\right|}{T_{i}},$$

where n = size of the dataset, $P_{i}$ = estimated results, and $T_{i}$ = experimental results.

### 2.3. SHAP Analysis

This research also determined global feature impacts and analyzed feature relations with CM, using a game theory approach known as SHAP [64]. SHAP analysis enhances the expandability of the suggested model. In this method, each case prediction is proved by computing all impact-considered characteristics, using SHapley values derived from the coalition game theory. Each feature’s impact on the SHapley value is somewhat averaged over all feasible combinations. The values of SHAP are directly proportional to the impact of characteristics. The mean of each input’s SHAP value is used to determine the global effect of each feature. These values are then arranged in order of descending significance, followed by the charting of SHAP values. On the SHAP plot, the SHAP value for each raw ingredient is represented by a single point. The X and Y axes show SHAP values and the significance of a feature, respectively. On the Y axis, its higher placement indicates the greater effect of the characteristic on the output, and a color gradient from light to dark is utilized to illustrate its significance. The interaction between characteristics and their effect on the outcomes is illustrated by SHAP plots with a color scheme that indicates feature interaction. This strategy provides greater information than conventional partial dependency graphs [65]. The $\phi^{j}\left(f\right)$ is the allotted impact for an ingredient impact, summed for the model’s outcome $f\left(x_{i}\right)$ to obtain the likely feature patterns [66]. The $\phi^{j}\left(f\right)$ is determined by Equation (4) as follows:

(4) $$\phi^{j}\left(f\right)=\sum_{S\subseteq \left\{x^{1},\ldots ..,x^{p}\right\}/\left\{x^{j}\right\}}\frac{\left|S\right|!\left(p-\left|S\right|-1\right)!}{p!}\left(f\left(S⊔\left\{x^{j}\right\}\right)-f\left(S\right)\right)$$

where

𝑆 = the ingredient subset,
$x_{j}$ = ingredient 𝑗
𝑝 = the ingredient number in the model.

In this technique, the significance of a feature is determined by measuring and estimating errors, while using a fixed feature value. Consideration is given to the estimated error sensitivity to allocate weight to the ingredient importance, while affecting its value. Moreover, SHAP illustrates the performance of the trained ML model. SHAP considers a different feature designation strategy, namely the linear addition of inputs, to provide an explainable model based on the model’s conclusion. For instance, a model with input parameters $x_{i}$, where i is in the range from 1 to k, and k represents the quantity of input parameters, and where $h\left(x_{s}\right)$ represents a description model with $x_{s}$ as a simple input, Equation (5) is used to illustrate an original model f(x).

(5) $$f\left(x\right)=h\left(x_{s}\right)=\emptyset_{0}+\sum_{i=1}^{p}\emptyset_{i}x_{s}^{i}$$

where

p = the input feature number
$\emptyset_{0}$ = the constant.

**Figure 6:**
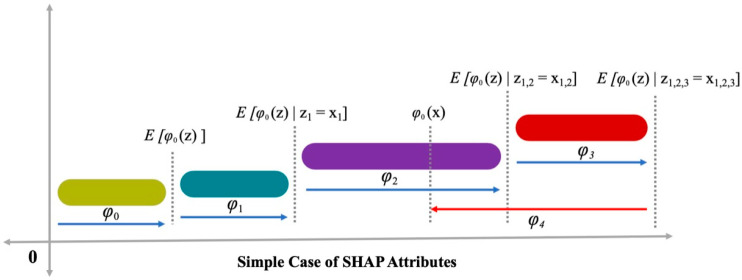
Attributes of SHAP [68].

In addition, $x=m_{x}\left(x_{s}\right)$, i.e., the mapping function is interlinked with both x and $x_{s}$ input factors. Lundberg and Lee [67] provided Equation (5), where $h\left(x_{s}\right)$, i.e., the estimation value, was increased by $\emptyset_{0},\;\emptyset_{1},\;\mathrm{and}\;\emptyset_{3}$ terms and a drop of $\emptyset_{4}$ in $h\left(x_{s}\right)$ was also identified (see Figure 6). A solution with a single value to Equation (5) incorporates three advantageous features, namely local accuracy, reliability, and missingness. Reliability verifies that no attribute decrease was assigned to the appropriate feature in a modification to a more influential feature. For missingness, it is established that missing features have no significant value; therefore, $\emptyset_{i}=0$ is applied by $x_{s}^{i}=0$. For local accuracy, it is established that the sum-up for attributing features as an output function includes a model that needs to match output f to $x_{s}$ as the simplified input. $x=m_{x}x_{s}$ signifies the achievement of local precision.

## 3. Results and Analysis

### 3.1. Compressive Strength Models

#### 3.1.1. Gradient Boosting Regressor Model

**Figure 7:**
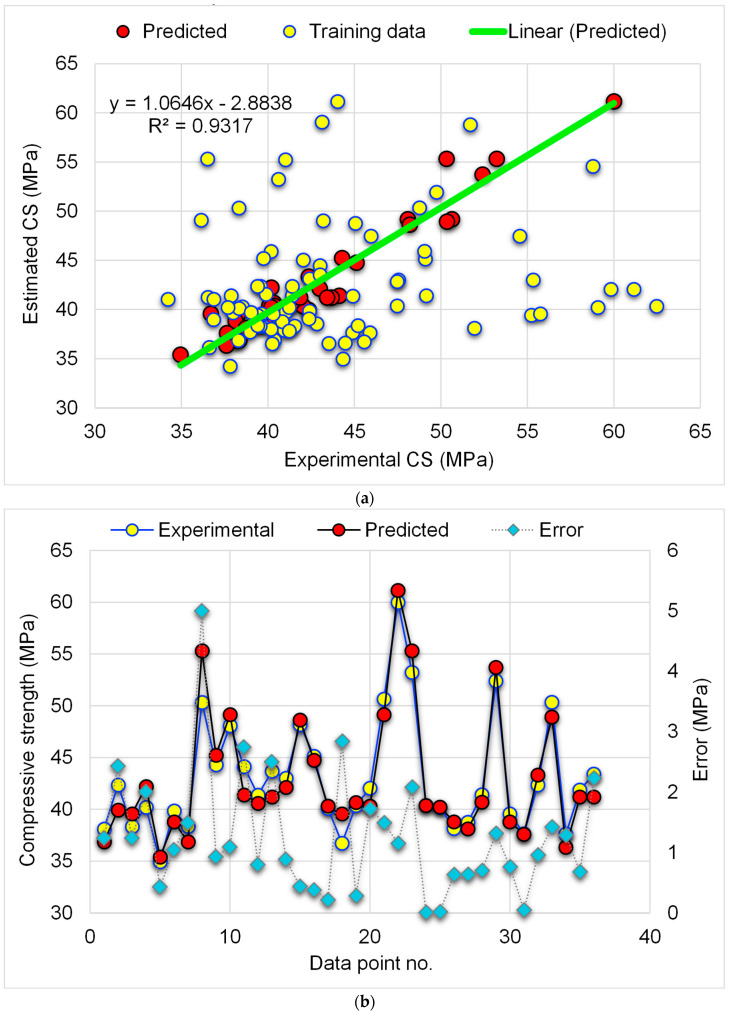
GBR-CS model: (**a**) correlation amongst experimental and predicted CS; (**b**) distribution of experimental and predicted CS and error values.

Figure 7 illustrates the results of the GBR model for estimating the WGP-based CM’s CS. Figure 7a depicts the association among experimental and projected CS. The GBR model forecasted CS with a high level of precision and little variance between the experimental and predicted results. The R^2^ value of 0.93 indicates that the GBR strategy for estimating the CS of WGP-based CM is exact, and the experimental and predicted results are in excellent agreement. Figure 7b depicts the experimental, predicted, and divergent value (errors) dispersion for the GBR method. The error value distribution ranged from 0.01 MPa to 5.0 MPa, with a mean of 1.25 MPa. In addition, the proportionate dispersion of errors was examined, and it was determined that 47.2% of the values were lower than 1 MPa, 30.6% fell among 1–2 MPa, and 22.2% were higher than 2 MPa. The division of divergent data (errors) indicates that the GBR approach predicted the CS of WGP-based CM accurately.

#### 3.1.2. Random Forest Regressor Model

**Figure 8:**
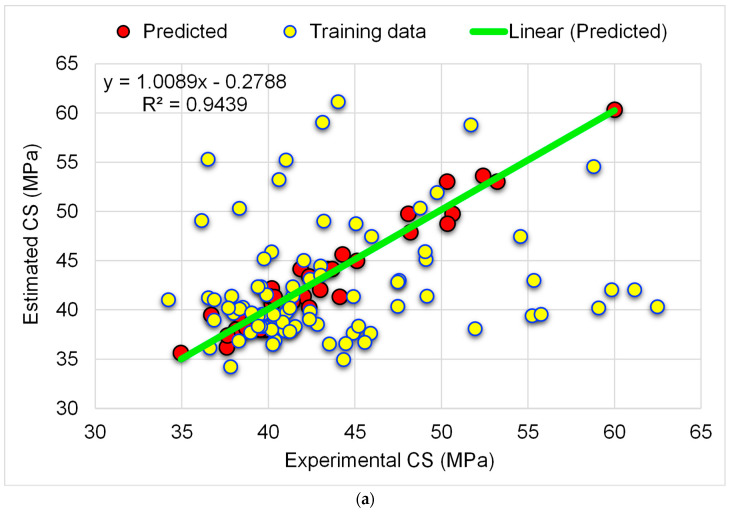

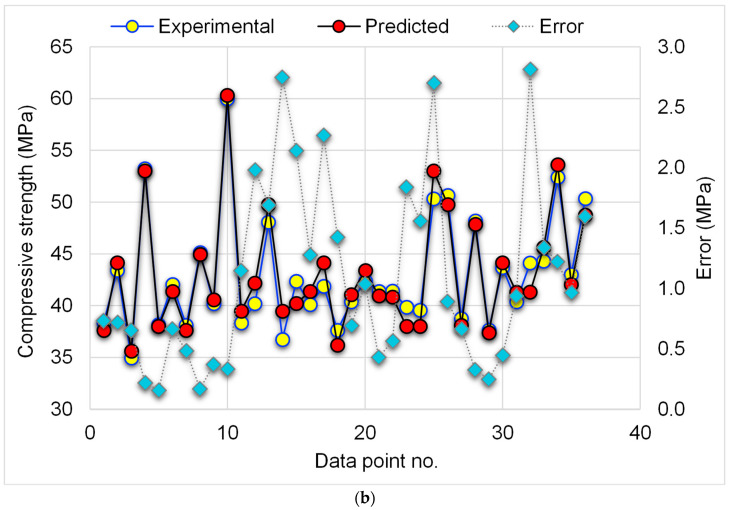
RFR-CS model: (**a**) correlation amongst experimental and predicted CS; (**b**) distribution of experimental and predicted CS and error values.

Figure 8 displays the results of the RFR approach used to forecast the CS of the WGP-based CM. The correlation among experimental and estimated CS can be observed in Figure 8a. Compared to the GBR model utilized in the current research, the RFR method produced more accurate results and the smallest discrepancy between the experimental and anticipated findings. The R^2^ value of 0.94 for the RFR model is indicative of its greater accuracy. Figure 8b illustrates the distribution of experimental, estimated, and divergent values (error) using the RFR method. The least, average, and maximum errors were 0.16 MPa, 1.10 MPa, and 2.81 MPa, respectively. Analyzing the error value distribution revealed that 55.6% fell below 1 MPa, 30.6% fell between 1 and 2 MPa, and 13.9% exceeded 2 MPa. Consequently, the error distribution suggested that the RFR model is more exact than the GBR model. The RFR model is more accurate because, in the RFR training process, each tree produces regression, and the forest with the highest number of votes is chosen as the model.

### 3.2. Flexural Strength Models

#### 3.2.1. Gradient Boosting Regressor Model

**Figure 9:**
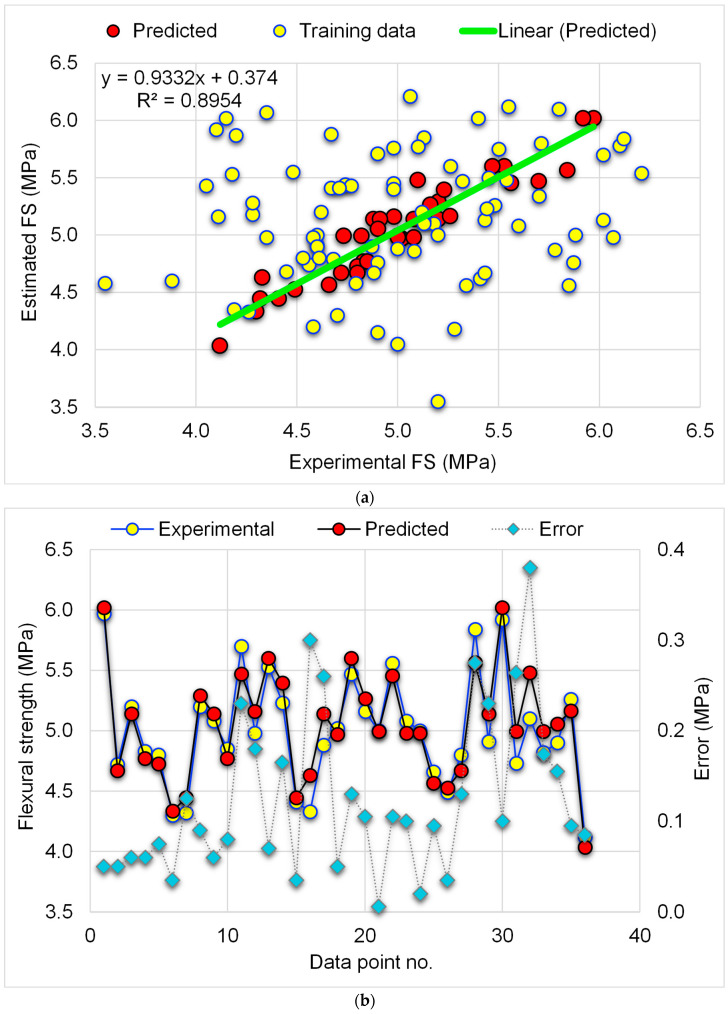
GBR-FS model: (**a**) correlation amongst experimental and predicted FS; (**b**) distribution of experimental and predicted FS and error values.

Figure 9 depicts the findings of the GBR approach for determining the FS of the WGP-based CM. The link between experimental data and expected results is depicted in Figure 9a. The GBR approach predicted FS with a satisfactory degree of precision and less variation among experimental and estimated FS. The R^2^ value of 0.90 suggests that the GBR technique for predicting the FS of WGP-based CM is satisfactory, with good agreement between the experimental and anticipated findings. Figure 9b displays the dispersion of experimental, anticipated, and divergent values (errors) using the GBR approach. The distribution of error values varied from 0.01 MPa to 0.38 MPa, with a mean of 0.12 MPa. Furthermore, the proportional dispersion of error values was investigated, and it was found that 50% of the errors were lower than 0.1 MPa, 30.6% were among 0.1–0.2 MPa, and 19.4% were larger than 0.2 MPa. The distribution of divergent data (errors) suggests that the GBR technique effectively anticipated the FS of WGP-based CM.

#### 3.2.2. Random Forest Regressor Model

**Figure 10:**
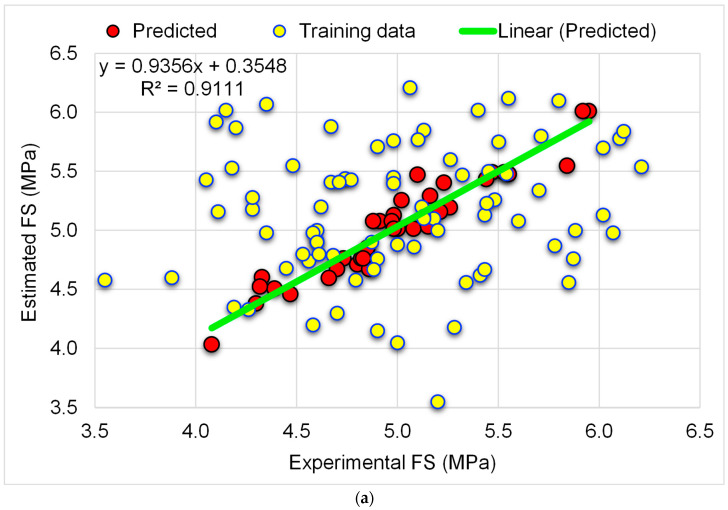

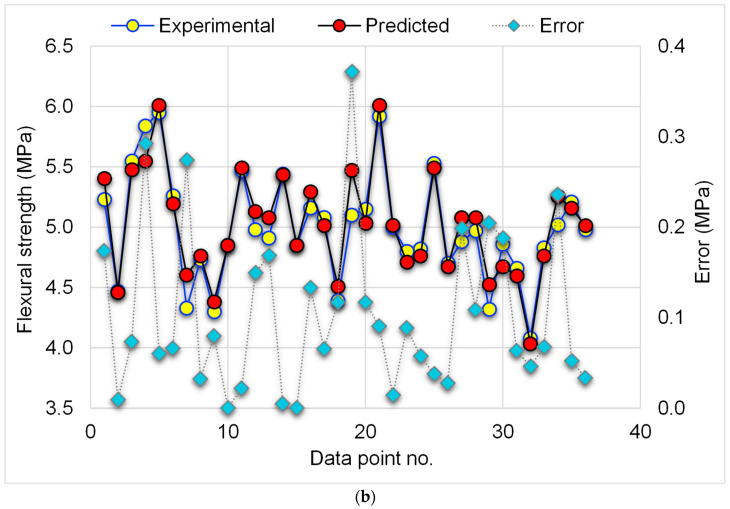
RFR-FS model: (**a**) correlation amongst experimental and predicted FS; (**b**) distribution of experimental and predicted FS and error values.

The findings of the RFR technique used to anticipate the FS of the WGP-based CM are shown in Figure 10. Figure 10a represents the link among experimental and predicted FS. The RFR technique yielded more accurate results and the least disparity between experimental and predicted outcomes. The RFR model’s R^2^ of 0.91 indicates its superior accuracy. Figure 10b depicts the RFR method’s distribution of experimental, estimated, and divergent values (errors). The average and maximum errors were determined to be 0.10 MPa and 0.37 MPa, respectively. The error value distribution indicated that 61.1% were less than 0.1 MPa, 25.0% were between 0.1 and 0.2 MPa, and 13.9% were greater than 0.2 MPa. As a result of the error distribution, the RFR model was shown to be more exact than the GBR model. It can be concluded that the RFR method is more accurate in predicting the CS and FS of WGP-based CM. However, the accuracy of the GBR model is also at an acceptable level. Hence, both models can be employed to assess the strength of CM incorporated with WGP.

### 3.3. Validation of Machine Learning Models

**Figure 11:**
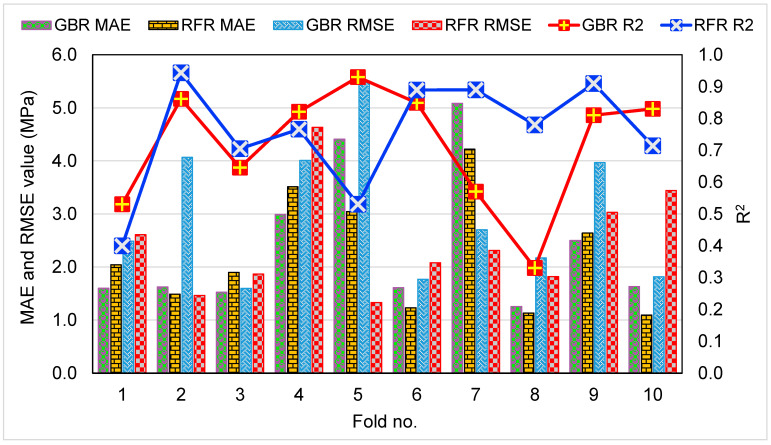
Results of k-fold assessment, indicating MAE, RMSE, and R^2^ for GBR and RFR models for the CS estimation.

**Figure 12:**
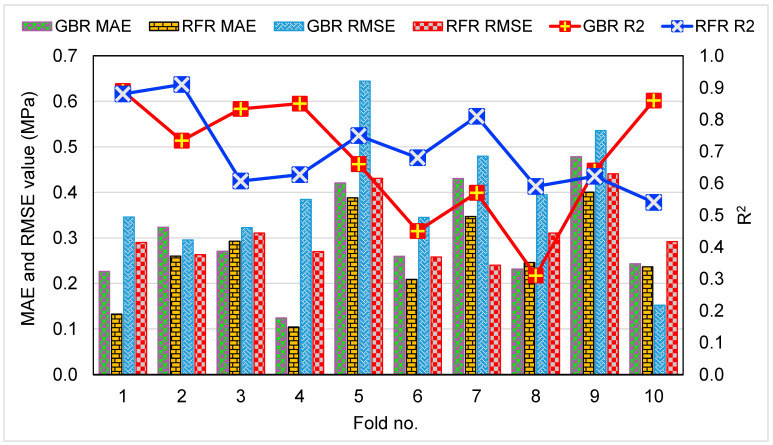
Results of k-fold assessment, indicating MAE, RMSE, and R^2^ for GBR and RFR models for the FS estimation.

The results of the error evaluations (MAE, MAPE, and RMSE) using Equations (1)–(3) above for both CS and FS estimation models are shown in Table 2. For the CS prediction, it was found that the MAE values for GBR and RFR were 1.254 MPa and 1.095 MPa, respectively. MAPE values for GBR and RFR were determined to be 2.90% and 2.60%, respectively. In addition, RMSE values for GBR and RFR were calculated to be 1.597 MPa and 1.331 MPa, respectively. These assessments also indicated that the RFR approach is more precise than the GBR due to its lower error rate. Similarly, for the FS prediction, the MAE, MAPE, and RMSE values for the GBR model were 0.124 MPa, 2.50%, and 0.152 MPa, respectively, whereas MAE, MAPE, and RMSE values for the RFR model were 0.104 MPa, 2.10%, and 0.137 MPa, respectively. These errors also validated the higher precision of the RFR model in estimating the FS of CM incorporated with WGP. The results of computing R^2^, RMSE, and MAE to validate the k-fold method are provided in Table 3. Figure 11 and Figure 12 were created for the comparison of both ML methods’ k-fold analyses for the CS and FS prediction, respectively. For the CS estimation, the range of MAE values for the GBR technique was 1.25 MPa to 5.09 MPa, with a mean of 2.42 MPa. The RFR model’s MAE ranged from 1.10 MPa to 4.22 MPa, with a mean of 2.23 MPa. In the same way, the average RMSE values for the GBR and RFR techniques were 3.01 MPa and 2.46 MPa, respectively. However, the average R^2^ values for GBR and RFR were 0.72 and 0.75, respectively. Comparatively, the RFR model with the lowest errors and the greater R^2^ is the more accurate in predicting the CS of WGP-based CM. Similar findings were noticed for the FS prediction models. The average MAE, RMSE, and R^2^ values for the GBR model were 0.30 MPa, 0.39 MPa, and 0.68, respectively, whereas for the RFR model, the average MAE, RMSE, and R^2^ values were 0.26 MPa, 0.31 MPa, and 0.70, respectively. The assessment of these errors and R^2^ values from the k-fold approach also confirmed the higher accuracy of the RFR model. However, the precision of the GBR model is also satisfactory. Hence, both GBR and RFR models might be employed to assess the CS and FS of CM with higher accuracy.

### 3.4. Impact of Raw Ingredients

**Figure 13:**
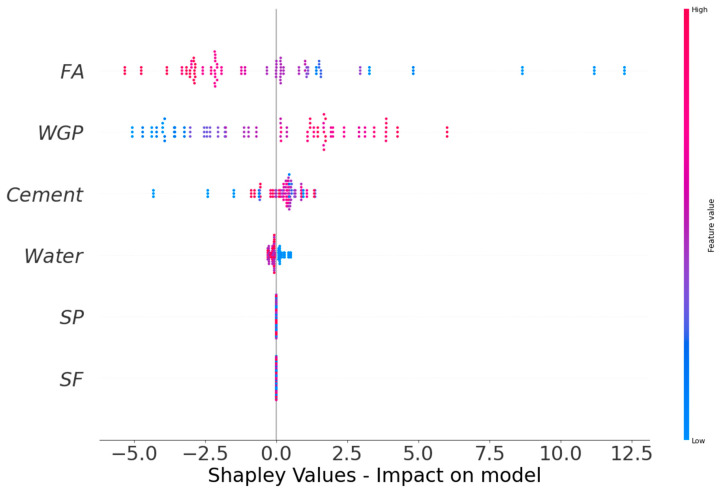
SHAP plot, indicating the impact of raw ingredients on CS and FS.

In this research, the effect of raw materials on the CS and FS performance of CM was investigated. The SHAP tree explainer is employed in the entire dataset to provide a more detailed explanation of the global feature effects by including local SHAP explanations. Figure 13 illustrates the results of the violin SHAP plot for all raw materials regarding the CS and FS of WGP-based CM. In this graph, each parameter value is characterized by a separate hue, and the resultant SHAP value on the x axis shows a raw ingredient’s contribution. FA is a raw material that has a greater impact on strength, as observed by the larger negative association between this feature and the CS of WGP-based CM (more red dots on the negative side). This implies that a rise in FA amount would likely lead to a decline in strength. It was established that the influence of WGP is more favorable (more red dots on the positive side), indicating that as WGP content increases, the material strength improves. However, a negative effect has also been found, suggesting that utilizing WGP in excess of the optimal quantity may reduce the strength. The effect of cement on the CS and FS was likewise shown to be more favorable, indicating that a larger cement fraction raises the strength. On the other hand, the influence of water on the CS and FS was shown to be more negative, suggesting that the water amount must be maintained low in order to attain a greater material strength. Due to the lack of input value variation in the employed dataset, it was concluded that the effect of SF and SP was unclear. Using a larger dataset with a broader variety of input characteristics may result in improved associations.

**Figure 14:**
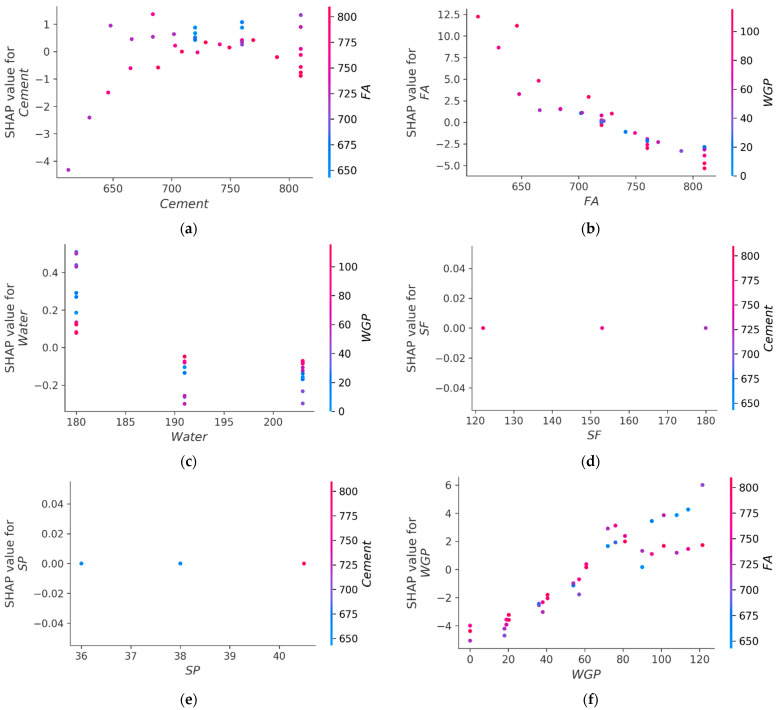
Interaction of raw ingredients and their impact on CS and FS: (**a**) cement; (**b**) fine aggregate; (**c**) water; (**d**) silica fume; (**e**) superplasticizer; (**f**) waste glass powder.

Figure 14 depicts the correlations between raw ingredients and their influence on the CS and FS of WGP-based CM. Figure 14a depicts the interaction of cement. The graph illustrates that as the cement amount grows, the material strength increases and mostly interacts with the FA. In contrast, increased FA levels have a detrimental effect on CS and FS (Figure 14b) and interact mostly with WGP. In addition, as shown in Figure 14c, water mostly interacts with WGP, and raising its value has a detrimental effect on its strength. Therefore, the water content should be reduced to increase its strength. Figure 14d,e demonstrate that the influence of SF and SP was uncertain based on the employed dataset, due to the lower variation in quantities for SF and SP. The incorporation of WGP into CM was shown to be advantageous (see Figure 14f). Among the possible causes are the filler effect and the pozzolanic property of WGP. The filler effect decreases porosity, resulting in a compact and dense matrix. The larger concentration of SiO_2_ in the chemical composition of glass reacts with Ca(OH)_2_ in the matrix to create a thick C-S-H gel, hence improving the performance of the material [69,70]. Utilizing WGP up to the optimum amount will assist in enhancing the strength of CM. Therefore, WGP may be utilized in the range of 80 to 120 kg/m^3^ to increase material strength. Moreover, WGP interacts primarily with the FA, among other input characteristics. This indicates that the use of WGP as a substitute for FA may result in greater strength compared to its usage as a cement replacement. It is crucial to note that these results are dependent on the kinds of raw materials and the size of the dataset studied in this research. Utilizing varied input settings and data samples could result in distinct outputs.

## 4. Discussions

Globally, a substantial quantity of GW is produced, most of which is discarded in landfills, posing health and environmental risks [23]. Furthermore, CM is the most common construction material, but its overuse depletes natural resources and generates CO_2_. GW has the possibility to be used as a partial substitute for fine aggregate and cement in CM, which is an environmentally favorable technique. Therefore, the use of GW in CM will decrease environmental impacts through the elimination of waste, preservation of natural resources, and reduction in CO_2_ emissions. Using ML-based modeling and SHAP analysis techniques, this research aimed to expand knowledge about the application of WGP in CM. This work estimated the CS and FS of WGP-based CM using GBR and RFR ML techniques. The accuracy of each technique was evaluated to ascertain which is the most exact predictor. Compared to the GBR method, with an R^2^ of 0.93 and 0.89 for CS and FS estimation, respectively, the RFR method produced more accurate results, with an R^2^ of 0.94 and 0.91 for CS and FS prediction, respectively. The disparity between the experimental and anticipated outcomes (errors) further substantiated the superior accuracy of the RFR approach. Compared to the GBR models, the experimental and estimated results for the RFR models showed good agreement, as demonstrated by the error analysis. In predicting the strength of CM, previous research has similarly demonstrated that the RFR method is more exact than the GBR method [61,71,72].

**Figure 15:**
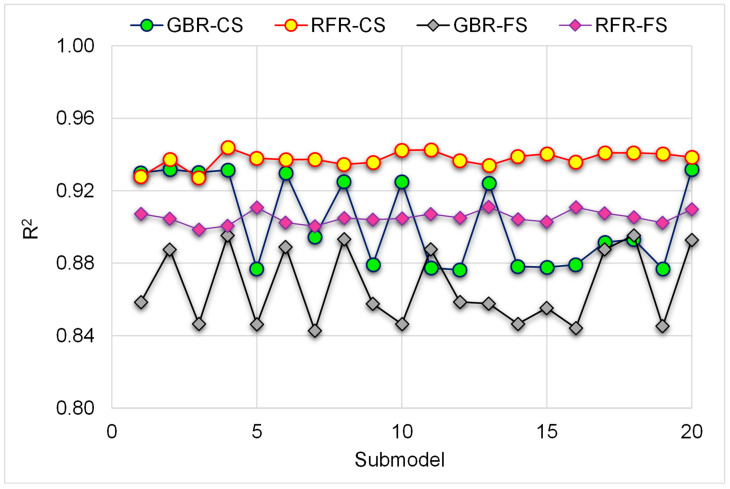
Distribution of R^2^ for the utilized ML models.

Moreover, the accuracy of both models was examined using statistical and k-fold approaches. When the degree of divergence (MAE, MAPE, and RMSE) is low and R^2^ is high, a model is more exact. Nevertheless, defining and recommending the optimal ML method for predicting attributes in various study areas is difficult, since the accuracy of an ML methodology is primarily reliant on the size of inputs and data samples used to execute algorithms [61]. Ensemble ML methods repeatedly employ the weak learner by developing sub-models that are trained on the data sample and tweaked to enhance the R^2^ value, thus resulting in more accurate outputs than the individual ML models. Figure 15 shows the dispersal of R^2^ for the GBR and RFR sub-models. The R^2^ for GBR-CS sub-models ranged from 0.876 to 0.932, with a mean of 0.903. In addition, the R^2^ for the RFR-CS sub-models varied between 0.927 and 0.944, with an average of 0.938. Likewise, the average R^2^ values for the GBR-FS and RFR-FS sub-models were 0.866 and 0.905, respectively. These findings reveal that the RFR sub-models are more precise than the GBR sub-models. Additionally, a SHAP analysis is conducted to investigate the interaction and influence of raw materials on the CS and FS of WGP-based CM. FA was demonstrated to be a very effective raw material, exhibiting a stronger negative association with the material’s strength. However, WGP was found to have a greater positive impact on the strength of CM. While WGP has been demonstrated to have beneficial effects, there is evidence that exceeding the optimal dosage may have negative consequences on performance. In addition, the impact of cement on CS and FS was shown to be more beneficial, indicating that strength increases with increasing cement content. However, due to the lack of variation in SF and SP in the data sample, their effect was ambiguous, and larger datasets with more input features may result in better associations.

## 5. Conclusions

This research aimed to employ experimental data to develop machine learning (ML)-based models to evaluate the compressive strength (CS) and flexural strength (FS) of cement mortar (CM) that contained waste glass powder (WGP). Two types of ensemble ML approaches, including gradient boosting regressor (GBR) and random forest regressor (RFR), were used to forecast the CS and FS. Moreover, SHapley Additive ExPlanations (SHAP) analysis was carried out to study the impact of raw ingredients on the strength of CM. This research reached the following conclusions:

- It was determined from the modeling methods that the GBR models had a satisfactory degree of precision, with an R^2^ of 0.93 and 0.89 for CS and FS prediction, respectively, while the RFR models had a higher degree of precision, with an R^2^ of 0.94 and 0.91 for CS and FS prediction, respectively.
- The average variation between predicted and experimental CS (error) in GBR and RFR models was determined to be 1.25 MPa and 1.10 MPa, respectively. Similarly, the average error values in predicting the FS of CM in the GBR and RFR models were 0.12 MPa and 0.10 MPa, respectively. These errors also confirmed the acceptable precision of the RFR models and higher accuracy of the RFR models in forecasting the strength of WGP-based CM.
- The SHAP study revealed that fine aggregate (FA) was a crucial raw material, with a higher negative correlation to the material’s strength. WGP and cement had a stronger favorable impact on CM’s strength. Due to the deficiency of variance in silica fume (SF) and superplasticizer (SP) in the data set, the effect of SF and SP was unclear.
- New techniques, such as ML-based modeling and SHAP analysis, will aid the building industry by fostering the advancement of fast and economical ways of determining material properties and the impact of raw ingredients.

This study employed data for which experimental work was performed in a controlled environment (laboratory). It is suggested that in future studies, actual on-site conditions, such as humidity, temperature, curing, etc., need to be incorporated during the modeling phase to examine their impact on the material’s strength.

## Figures and Tables

**Table 1:** Descriptive statistical analysis of input and output variables.

Parameter	Cement (kg/m^3^)	FA (kg/m^3^)	Water (kg/m^3^)	SF (kg/m^3^)	SP (kg/m^3^)	WGP (kg/m^3^)	CS (MPa)	FS (MPa)
Mean	732.51	735.62	191.33	151.67	38.17	61.65	43.48	5.00
Standard error	4.96	5.04	0.87	2.20	0.17	3.36	0.56	0.05
Median	722.00	729.00	191.00	153.00	38.00	60.75	41.40	4.98
Mode	810.00	810.00	203.00	122.00	40.50	0.00	40.20	4.90
Standard deviation	53.62	54.51	9.43	23.80	1.85	36.34	6.09	0.56
Minimum	612	612	180	122	36	0	34.24	3.55
Maximum	810	810	203	180	40.5	121.5	62.46	6.21

**Table 2:** Error evaluations of the developed models using statistical checks.

ML Method	CS Model			FS Model		
	MAE (MPa)	MAPE (%)	RMSE (MPa)	MAE (MPa)	MAPE (%)	RMSE (MPa)
GBR	1.254	2.90	1.597	0.124	2.50	0.152
RFR	1.095	2.60	1.331	0.104	2.10	0.137

**Table 3:** Results of k-fold assessment.

K-fold	CS						FS					
	GBR			RFR			GBR			RFR		
	MAE	RMSE	R^2^	MAE	RMSE	R^2^	MAE	RMSE	R^2^	MAE	RMSE	R^2^
1	1.60	2.48	0.53	2.04	2.61	0.40	0.23	0.35	0.89	0.13	0.29	0.88
2	1.62	4.06	0.86	1.49	1.46	0.94	0.32	0.30	0.73	0.26	0.26	0.91
3	1.52	1.60	0.65	1.90	1.87	0.70	0.27	0.32	0.83	0.29	0.31	0.61
4	2.99	4.01	0.82	3.51	4.63	0.77	0.12	0.38	0.85	0.10	0.27	0.63
5	4.41	5.48	0.93	3.04	1.33	0.53	0.42	0.64	0.66	0.39	0.43	0.75
6	1.61	1.77	0.85	1.23	2.08	0.89	0.26	0.34	0.45	0.21	0.26	0.68
7	5.09	2.70	0.57	4.22	2.31	0.89	0.43	0.48	0.57	0.35	0.24	0.81
8	1.25	2.17	0.33	1.13	1.82	0.78	0.23	0.39	0.31	0.25	0.31	0.59
9	2.50	3.97	0.81	2.64	3.03	0.91	0.48	0.54	0.64	0.40	0.44	0.62
10	1.63	1.82	0.83	1.10	3.44	0.71	0.24	0.15	0.86	0.24	0.29	0.54

## Data Availability

The data used in this research have been properly cited and reported in the main text.
